# Differences in the risk of frailty based on care receipt, unmet care needs and socio-economic inequalities: A longitudinal analysis of the English Longitudinal Study of Ageing

**DOI:** 10.1016/j.tjfa.2025.100012

**Published:** 2025-04

**Authors:** David R Sinclair, Asri Maharani, Andrew Clegg, Barbara Hanratty, Gindo Tampubolon, Chris Todd, Raphael Wittenberg, Terence W O'Neill, Fiona E Matthews

**Affiliations:** a National Institute for Health and Care Research (NIHR) Older People and Frailty Policy Research Unit, Population Health Sciences Institute, Newcastle University, Newcastle upon Tyne, NE1 7RU, UK; b Division of Nursing, Midwifery and Social Work, School of Health Sciences, Faculty of Biology, Medicine and Health, The University of Manchester, Manchester, M13 9PL, UK; c Academic Unit for Ageing and Stroke Research, Bradford Institute for Health Research, School of Medicine, University of Leeds, Leeds, LS2 9JT, UK; d Global Development Institute, School of Environment, Education and Development, Faculty of Humanities, University of Manchester, Manchester, M13 9PL, UK; e National Institute for Health and Care Research (NIHR) Older People and Frailty Policy Research Unit, School of Health Sciences, Faculty of Biology, Medicine and Health, The University of Manchester, Manchester, M13 9PL, UK; f National Institute for Health and Care Research (NIHR) Older People and Frailty Policy Research Unit, Care Policy and Evaluation Centre, The London School of Economics and Political Science, London, WC2A 2AE, UK; g University of Hull, Cottingham Road, Hull, HU6 7RX, UK

**Keywords:** Prefrail, ELSA, Multistate model, Healthy ageing, Unmet need for care

## Abstract

**Background:**

The older population is increasingly reliant on social care, especially those who are frail. However, an estimated 1.5 million people over 65 in England have unmet care needs. The relationship between receiving care, or receiving insufficient care, and changes in frailty status remains unclear.

**Objectives:**

To investigate the associations between care receipt (paid or unpaid), unmet care needs, frailty status, and mortality.

**Design:**

We used multistate models to estimate the risk of increasing or decreasing levels of frailty, using English Longitudinal Study of Ageing (ELSA) data. Covariates included age, gender, wealth, area deprivation, education, and marital status. Care status was assessed through received care and self-reported unmet care needs, while frailty status was determined using a frailty index.

**Participants:**

15,003 individuals aged 50+, using data collected over 18 years (2002–2019).

**Results:**

Individuals who receive care are more susceptible to frailty and are less likely to recover from frailty to a less frail state. The hazard ratio of males receiving care transitioning from prefrailty to frailty was 2.1 [95 % CI: 1.7–2.6] and for females 1.8 [1.5–2.0]. Wealth is an equally influential predictor of changes in frailty status: individuals in the lowest wealth quintile who *do not* receive care are as likely to become frail as those in the highest wealth quintile who *do* receive care. As individuals receiving care (including unpaid care) are likely to be in poorer health than those who do not receive care, this highlights stark inequalities in the risk of frailty between the richest and poorest individuals. Unmet care needs were associated with transitioning from prefrailty to frailty for males (hazard ratio: 1.7 [1.2–2.4]) but not for females.

**Conclusions:**

Individuals starting to receive care (paid or unpaid) and people in the poorest wealth quintile are target groups for interventions aimed at delaying the onset of frailty.

## 1 Background

As populations age, older people will account for an increasing proportion of health and social care services [1]. Frailty is a valuable measure of the health of older people, characterised by a decline in physiologic and cognitive reserves and function, which leads to an increased vulnerability to stressors [2]. Frailty is associated with adverse outcomes, including higher mortality rates and the use of health and social care services [[3], [4], [5], [6]]. Estimates of frailty prevalence in England vary by metric and age groups, but recent estimates range from 3 to 14 % of older people [[7], [8], [9]], with a further 10–12 % being prefrail [7,8].

Many older people with frailty receive some social care. In the UK, this is provided by both public and private expenditure, as well as by unpaid care from family and friends. Despite this, an estimated 1.5 million people over 65 have unmet care needs [10]. Unmet care needs exist where care provision does not meet the requirements of the care recipient, whether it is paid or unpaid care. Inequalities in unmet care needs may result from social policies, family relations and societal structures [11]. Previous research has found unmet care needs to be associated with increased levels of mortality, hospitalisations, falls, injuries, stress and loneliness [[12], [13], [14], [15], [16], [17]]. They also report reduced levels of life satisfaction [13]. However, the evidence on the effect of unmet care needs on the risk of frailty is limited.

The extra annual cost to the healthcare system for each older person living with frailty is estimated to be £1200-£2100 (UK, 2013/14 reference costs) [18]. Furthermore, it has been estimated that the total at-home formal social care costs for England could be reduced by £4.4 million per annum (2021 costs) for every 1 % of robust people who are prevented from becoming frail [19]. By advancing our understanding of who is most at risk of frailty, we may be able to enhance the well-being of older individuals and generate substantial cost savings for the healthcare and social service industries.

This study aims to understand how care receipt, unmet care needs and socio-economic characteristics are associated with longitudinal health outcomes, as measured by frailty.

## 2 Methods

### 2.1 Study population

We used data from the English Longitudinal Study of Ageing (ELSA), a nationally representative prospective cohort study of people aged 50 and over in England [20]. ELSA surveys approximately 10,000 people every two years, collecting information on demographic, socio-economic, health and lifestyle characteristics. So far, ELSA has conducted 9 waves from 2002 to 2019. Participants are recruited randomly from private households (called core members) or by cohabiting with a core member (called partners). ELSA does not sample care home residents at baseline but participants who move into a care home remain eligible for subsequent waves. Mortality data is recorded by ELSA and linked data (Appendix A1). We used data from core members aged 50 and older.

### 2.2 Frailty

We measured frailty with a frailty index [21]. Following the frailty index described by Maharani et al. [22], we used sixty deficits in ELSA covering mobility, chronic diseases, cognitive ability, and sensory impairment (Appendix A2). We stratified frailty index scores into three categories using commonly defined cut points: robust (frailty index ≤ 0.08), prefrail (frailty index >0.08 and <0.25) and frail (frailty index ≥ 0.25) [[23], [24], [25]].

### 2.3 Care

We used two primary definitions of care, the first measuring receipt of care and the second recording unmet need for care.

Receipt of care was defined as receiving any help for a range of tasks, including Activities of Daily Living (ADLs), Instrumental Activities of Daily Living (IADLs) and mobility (Appendix A3). This encompasses both paid and unpaid help.

Unmet need for care is more difficult to measure. Two approaches are commonly used: self-reporting of inadequate care provision, and asking older people if care is provided for each reported ADL or IADL disability [26]. Both methods have weaknesses: the former is vulnerable to self-reporting bias, while the latter may underestimate unmet care, as it does not measure the sufficiency of care provided [27,28]. Here, we use the self-reported method, recording unmet need for care when participants said the care they received ‘sometimes’ or ‘hardly ever’ met their needs (versus ‘always’ or ‘usually’ met their needs).

Both receipt of care and unmet need for care were recorded as binary variables.

### 2.4 Covariates

Frailty is not solely distributed by age and gender in the older population, with studies finding associations with socio-economic and demographic factors. Four socio-economic covariates were analysed in our models: wealth, educational attainment, marital status (self-reported measures) and area deprivation (derived from participant postcode).

Wealth was defined as the net total wealth of the respondent's ‘benefit unit’, where a benefit unit is a single adult or a married/cohabiting couple, and any dependent children. Wealth was separated into quintiles, with quintile 1 having the least wealth and quintile 5 having the most wealth.

We categorised area deprivation using the English Index of Multiple Deprivation (IMD, the UK government's official measure of deprivation) [29]. IMD divides the country into areas of 1000–3000 people and orders them from the most deprived to the least deprived. The most deprived areas typically have lower employment rates, more crime and more barriers to housing and services. We stratified the IMD ranking into quintiles, with quintile 1 being the most deprived areas and quintile 5 being the least deprived areas. This ordering may seem counterintuitive compared to the wealth quintiles (as quintile 1 is used for the *least* wealthy but also the *most* deprived), however, it ensures that both variables are arranged from the least advantaged to the most advantaged individuals.

Educational attainment was stratified into lower than secondary school, secondary school, and college or higher, while marital status was divided into married and not married.

### 2.5 Statistical analysis

**Fig. 1:**
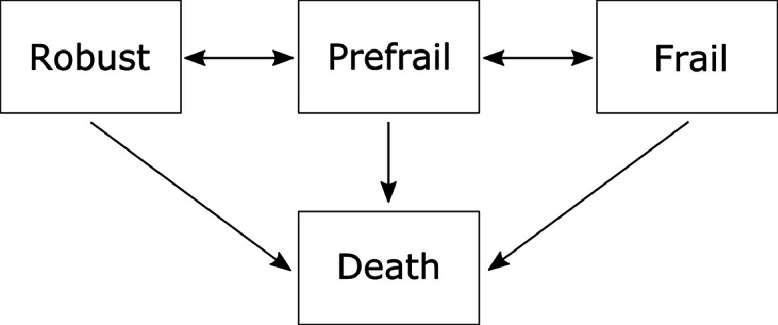
State transition diagram for all models. ELSA participants are tracked transitioning between the frailty states and death to determine the risk of each state transition. Socio-economic and demographic covariates adjusted the risk of transitioning between states.

The relationship between frailty and care receipt was modelled using multistate models with longitudinal data from ELSA waves 1–9. A state transition model was defined allowing individuals to move between robust, prefrail, frail and death states (Fig. 1). Bidirectional transitions were possible between adjacent frailty states. All states could unidirectionally transition to the (absorbing) death state. Individuals could also remain in the same state.

The risk of a person with a given covariate (e.g. receiving care) moving from one state to another in a given time period, compared to a person without the given covariate (e.g. does not receive care) is measured by a Hazard Ratio (HR). A hazard ratio greater than 1 indicates an increased risk of the transition occurring, whereas a hazard ratio less than 1 indicates a reduced risk of the transition occurring.

Transitions were possible during each model time step (∆*t* = 1 month). Some participants transition from robust-to-frail (or frail-to-robust) between consecutive ELSA waves; the model assumes they transition through the prefrail state during the time steps between waves. Although data may not be available to identify participants who transition back and forth between states multiple times between consecutive ELSA waves, the multistate model assumes these are possible when optimising the model fit.

The relationship between frailty and care receipt was investigated using age, wealth, area deprivation, and educational attainment as continuous covariates, as well as marital status as a binary covariate. For each model, one of these socio-economic covariates was adjusted alongside age and one measure of care status. Separate analyses were conducted for men and women due to their differences in frailty prevalence and mortality rates [[30], [31], [32], [33]].

Participants with no longitudinal data were not included. We included 15,003 participants over 9 waves, with 9491 at baseline (ELSA has recruited new participants in most waves to maintain population representativeness). Model selection was conducted with the Bayesian Information Criterion (BIC). Models were fitted using the *msm* package (version 1.6.9) in R (version 4.2.1) [34,35].

Sensitivity analyses adjusting the definition of care receipt, unmet need for care and participant date of birth and death were conducted (Appendix A4).

### 2.6 Ethics statement

Ethical approval for all ELSA waves were obtained from the National Research and Ethics Committee. Participants gave full informed written consent to participate in the study. Separate ethical approval for the current analysis was not required.

## 3 Results

Table 1 shows the characteristics of the ELSA wave 1 (i.e., baseline) population. There was a greater proportion of women than men (55.0% vs 45.0 %), with 69.6 % of the baseline population aged 50–70 years. Participants were more likely to come from less deprived areas (47 % in quintiles 4 and 5) and be married (67.6 %). Educational attainment was split: 40.0 % had less than a secondary school education while 43.4 % had college or higher education. One in five (21.1 %) received care, with 2.6 % reporting unmet need for care.

**Table 1:** Characteristics of ELSA wave 1 population.

Characteristic		n	(%)
All		9491	
Gender			
	Male	4268	45.0
	Female	5223	55.0
Age			
	50–54	1801	19.0
	55–59	1880	19.8
	60–64	1466	15.4
	65–69	1460	15.4
	70–74	1209	12.7
	75–79	859	9.1
	80–84	593	6.2
	85–89	209	2.2
	90+	14	0.1
Deprivation quintile			
	1 (Most deprived)	1362	14.4
	2	1735	18.3
	3	1935	20.4
	4	2227	23.5
	5 (Least deprived)	2232	23.5
Marital status			
	Not married	3071	32.4
	Married	6418	67.6
Education			
	Lower than secondary school	3797	40.0
	Secondary school	1574	16.6
	College or higher	4120	43.4
Wealth quintile			
	1 (Least wealthy)	1749	18.7
	2	1772	19.0
	3	1851	19.8
	4	1950	20.9
	5 (Most wealthy)	2009	21.5
Receipt of care			
	No	7488	78.9
	Yes	1997	21.1
Unmet need for care			
	No	9241	97.4
	Yes	245	2.6

Care receipt (Table 2a) was associated with an increased risk of transitioning from robust-to-prefrail states (HR: males 2.1 [1.7–2.6], females 1.8 [1.5–2.0]) and decreased risk of the reverse transition (males: 0.5 [0.4–0.6], females: 0.5 [0.4–0.5]). Similarly, receiving care was associated with an increased risk of transitioning from prefrail-to-frail (males: 2.6 [2.3–2.9], females: 2.3: [2.1–2.5]) and decreased risk of frail-to-prefrail (males: 0.7 [0.6–0.8], females: 0.6 [0.6–0.7]).

**Table 2:** State transition hazard ratios and 95 % confidence intervals [CI] for receipt of care, wealth and age covariates. Results are split by gender. Wealth is categorised into quintiles, with quintile 1 being the least wealthy. It was not possible to accurately constrain the robust-death transition for males in receipt of care due to the small number of recorded transitions.

Male	Age [CI]		Receive care [CI]		Wealth [CI]	
Robust–Prefrail	1.004	[1.004–1.005]	2.09	[1.70–2.58]	0.87	[0.85–0.90]
Robust–Death	1.007	[1.005–1.009]	0.17	[-]	0.81	[0.70–0.94]
Prefrail–Robust	0.997	[0.996–0.997]	0.47	[0.39–0.55]	1.16	[1.13–1.20]
Prefrail–Frail	1.003	[1.003–1.004]	2.56	[2.27–2.90]	0.82	[0.78–0.85]
Prefrail–Death	1.007	[1.006–1.008]	1.10	[0.84–1.45]	0.95	[0.88–1.04]
Frail–Prefrail	0.999	[0.999–1.000]	0.65	[0.55–0.77]	1.08	[1.01–1.16]

Female						

Robust–Prefrail	1.003	[1.003–1.004]	1.75	[1.50–2.04]	0.88	[0.86–0.90]
Robust–Death	1.008	[1.006–1.010]	0.88	[0.14–5.59]	0.97	[0.81–1.16]
Prefrail–Robust	0.996	[0.996–0.996]	0.48	[0.42–0.54]	1.12	[1.09–1.15]
Prefrail–Frail	1.003	[1.003–1.004]	2.26	[2.05–2.48]	0.83	[0.80–0.86]
Prefrail–Death	1.009	[1.008–1.010]	1.17	[0.87–1.57]	0.84	[0.76–0.94]
Frail–Prefrail	0.999	[0.998–0.999]	0.64	[0.56–0.74]	1.18	[1.12–1.24]
Frail–Death	1.006	[1.005–1.006]	1.13	[0.92–1.40]	1.01	[0.95–1.07]

Unmet need for care (Table 3) was not associated with any transition risk for females. However, for males, it was associated with a higher risk of transitioning from prefrail-to-frail (1.7 [1.2–2.4]).

**Table 3:** State transition hazard ratios and 95 % confidence intervals [CI] for unmet need for care, wealth and age covariates. Results are split by gender. Wealth is categorised into quintiles, with quintile 1 being the least wealthy.

Male	Age [CI]		Unmet care [CI]		Wealth [CI]	
Robust–Prefrail	1.005	[1.004–1.005]	0.76	[0.45–1.29]	0.87	[0.85–0.90]
Robust–Death	1.005	[0.998–1.012]	1.01	[0.12–8.74]	0.43	[0.14–1.35]
Prefrail–Robust	0.997	[0.996–0.997]	0.78	[0.46–1.32]	1.18	[1.14–1.22]
Prefrail–Frail	1.004	[1.004–1.005]	1.71	[1.21–2.42]	0.83	[0.80–0.87]
Prefrail–Death	1.002	[0.999–1.004]	1.03	[0.85–1.24]	1.01	[0.80–1.27]
Frail–Prefrail	0.998	[0.998–0.999]	1.02	[0.94–1.10]	1.05	[0.98–1.12]
Frail–Death	1.004	[1.003–1.004]	1.00	[0.96–1.03]	1.10	[1.04–1.17]

Female						

Robust–Prefrail	1.003	[1.003–1.004]	0.78	[0.58–1.06]	0.88	[0.86–0.90]
Robust–Death	1.004	[1.001–1.007]	1.03	[0.05–20.4]	0.76	[0.62–0.93]
Prefrail–Robust	0.996	[0.995–0.996]	0.78	[0.58–1.06]	1.13	[1.10–1.16]
Prefrail–Frail	1.004	[1.004–1.004]	1.00	[0.97–1.03]	0.84	[0.82–0.87]
Prefrail–Death	1.007	[1.003–1.011]	1.05	[0.06–17.0]	0.37	[0.16–0.88]
Frail–Prefrail	0.998	[0.998–0.999]	1.01	[0.97–1.05]	1.15	[1.10–1.21]
Frail–Death	1.004	[1.004–1.005]	0.99	[0.98–1.01]	1.13	[1.08–1.18]

Greater wealth was associated with a lower risk of frailty (i.e. robust-to-prefrail and prefrail-to-frail) and with increased recovery to less frail states (i.e. frail-to-prefrail and prefrail-to-robust) in both the care receipt and unmet need for care models. The only exception was that wealthy males with frailty were *not* more likely to recover to prefrailty than less wealthy males in the unmet care model. Wealth was the most important socio-economic covariate, providing the best fit for both the care receipt (Table 2) and unmet need for care models (Table 3), as measured by BIC. The transition hazard ratios for each of the models are included in the Appendix A5 and A6.

Wealth is associated with mortality, however, care receipt and unmet need for care are not. The risk of death is reduced with increased wealth for robust males and prefrail females in the care receipt model. In the unmet need for care model, increased wealth is associated with reduced risk of death for robust and prefrail females, but increased risk of death for males and females with frailty.

**Fig. 2:**
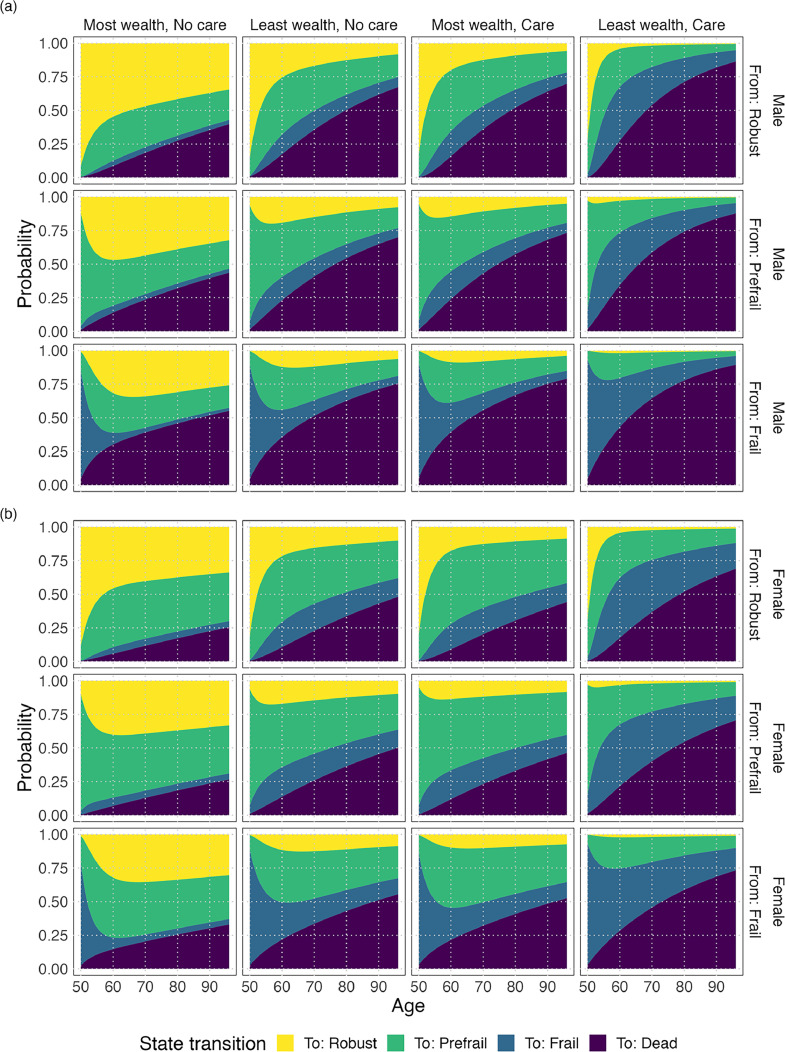
Transition probabilities from each frailty state to each frailty state and death from age 50–95 for (a) males and (b) females. Each row of plots indicates a person's currently occupied frailty state. Each column of plots indicates their wealth status and whether they receive care. For example, the top-left plot indicates the probabilities for a male who is robust, in the highest wealth quintile and does not receive care; the plot shows the probability of transitioning from robust to each of the other states in the next month for ages 50–95.

Both receipt of care and low wealth are strongly associated with changes in frailty status (Fig. 2 and Appendix A7). In nearly every instance, the risk of frailty for someone with low wealth (vs high wealth) matches the risk for someone receiving care (vs no care). The risk of increasing frailty state (or dying) for individuals in the lowest wealth quintile who do not receive care, is similar to the risk for individuals in the highest wealth quintile who do receive care (Appendix A5). The single difference in frailty risk for receiving care vs not receiving care, compared to high wealth vs low wealth, is that prefrail females who receive care are less likely to recover to the robust state (Appendix A7).

The results for models with the other covariates (educational attainment, area deprivation and marital status) using the care receipt model are included in Appendix A5 (a-d). As with the wealth model, care receipt increased the risk of greater frailty and reduced the risk of decreasing frailty when the other socio-economic covariates were considered.

After adjusting for receipt of care and age, higher educational attainment was associated with lower hazard ratios for increasing frailty and higher hazard ratios for prefrail-to-robust. It also increased the likelihood of recovery from frail-to-prefrail for females. The same associations existed for less deprived areas and marriage. Marriage was additionally associated with a lower risk of death for robust and prefrail males, but not females.

The unmet need for care models with each socio-economic covariate are shown in Appendix A6 (a-d). Unlike receipt of care, unmet need for care is not associated with many transitions at the 95 % confidence interval in any of the models. It is associated with an increased risk of transitioning from prefrail to frail for males, an association consistent across models with the four socio-economic covariates: wealth, deprivation, education, and marriage. In the deprivation model alone, unmet need for care is also associated with reduced risk of robust-to-prefrail and prefrail-to-robust.

Focusing on the socio-economic covariates in the unmet need for care models: higher educational attainment is associated with reduced risk of frailty and increased recovery from prefrail-to-robust for males and females. It is also associated with recovery from frail-to-prefrail for females and reduced risk of death for robust and prefrail females. Living in a less deprived area is similarly associated with a reduced risk of increased frailty and increased recovery to lower frailty states for males and females. Living in less deprived areas is further associated with a reduced risk of death for robust females and an increased risk of death for frail males and females. Marriage follows the same pattern as living in less deprived areas, except it is not associated with recovery to lower frailty states for females, nor increased risk of death for frail males.

The sensitivity analyses did not change the overall results (Appendix A4).

## 4 Discussion

This longitudinal analysis, spanning 18 years of data, suggests individuals who receive care (paid or unpaid) are more susceptible to frailty and are less likely to recover from frailty to a less frail state. Furthermore, it reveals that individuals with higher household wealth are less likely to develop frailty and more likely to recover to a less frail state than those with lower household wealth. Notably, household wealth is more strongly associated with frailty than other socio-economic factors, including area deprivation, education level, and marital status (as measured by each model's BIC).

Walsh et al. found that area deprivation was a more significant predictor of transitioning to frailty than ethnicity and living in an urban area using an electronic Frailty Index [32,36]. Our results find that household wealth is a better predictor than area deprivation. Like Walsh et al., we found that increased age and lower socio-economic status are associated with increased risk of becoming frail.

It is perhaps surprising that receiving care is associated with susceptibility to frailty and a reduced chance of recovering from frailty. It's not possible to ascertain from this analysis whether receiving care is a direct cause of the increased susceptibility to frailty or that it is indicative of a decline in health that presages frailty. Many studies have shown poor self-reported health to be associated with future adverse health outcomes (e.g. [[37], [38], [39], [40], [41]]). It's possible that someone recognising an individual's requirement for social care, whether that be the individual themselves, a family member or someone else, may similarly predict future poor health.

This study highlights two specific groups that provide an opportunity for targeted interventions to reduce the occurrence and progression of frailty and reduce the economic investment required to provide health and social care to people with frailty. These are individuals with lower wealth and individuals who are receiving any type of care. Such interventions might include implementing physical activity and nutritional interventions [42,43]. Identifying those who start receiving care could generate the most success, as these people are least likely to have increased their level of frailty and so may benefit most from a proactive intervention. Although identifying people who receive unpaid care may be more complex than using registers of formal home care provision, identifying only those who receive paid-for care risks exacerbating health inequalities, as prior studies suggest wealthier households are more likely to have paid-for care [[44], [45], [46]].

Receiving care is a greater indicator of a person's change in frailty state than having an unmet need for care. Unmet need for care was not associated with any transitions for females, although it was associated with an increased risk of prefrail males progressing to frailty. It is unclear whether the few associations of unmet need for care is a limitation of the survey data. The smaller number of ELSA participants reporting unmet need for care (wave 1: *n* = 245, 2.6 %) compared to receiving care (wave 1: *n* = 1997, 21.1 %) may have led to larger confidence intervals in the model output. The subjective nature of whether care needs are ‘always’, ‘usually’, ‘sometimes’ or ‘hardly ever’ met may also obscure any underlying relationship.

Our results suggest low wealth is an important predictor of frailty and of those less likely to recover from frailty. The strength of the association between wealth and changes in frailty is similar to that of receiving care (as shown in Fig. 2). As a person who receives care (including unpaid care from family/friends) is likely to be in poorer health than a person who does not receive care, this highlights strong inequalities in a person's risk of frailty between individuals with high and low wealth. The associations between wealth and frailty are present for both the received care and unmet care models and are consistent between males and females.

Low wealth is also a predictor for mortality, unlike receipt of care or unmet need for care. Counter-intuitively, greater wealth was associated with greater mortality for people with frailty in the unmet need for care model. Wealth may allow people to live a greater proportion of their lives in the robust and prefrail state, which may explain the decreased time spent in the frail state.

When considering alternative socio-economic covariates, we found a consistent pattern where greater socio-economic advantages benefit frailty-free health. Living in a less deprived area, more education and being married are all associated with reduced risk of frailty and increased recovery to lower frailty states. This agrees with previous studies which found lower frailty prevalence among those with socio-economic advantages [5,7,22,47,48].

A previous review identified that being male, having a low income, and experiencing more functional limitations are all associated with unmet needs [49]. This aligns with our finding that unmet care is associated with prefrailty in males, but not in females. Our data does not explain this gender difference. Future research which includes qualitative analysis could help explore any underlying mechanisms.

A prior study found that care receipt is associated with higher risk of unplanned hospital admission, independently of frailty status [50]. However no link was found between unmet care and unplanned hospitalisations. This supports our finding that older individuals who receive care should be a key focus of intervention efforts.

### 4.1 Limitations

We drew on a large, nationally representative data set, which has recorded participant data over 18 years with detailed information about their care receipt, socio-economic and demographic factors and frailty status, however, there are limitations to the analysis. Unmet need for care is non-trivial to measure; we used a subjective measure which is vulnerable to self-reporting bias. We also do not distinguish between paid and unpaid care receipt.

Despite the size of ELSA, there are a limited number of transitions between some states, such as people who die when they are robust. The number of ELSA participants reporting unmet need for care is also low in our main definition (2.6 %). These small numbers result in large confidence intervals for some hazard ratios.

ELSA effectively represents the socio-demographic characteristics of the older population in England. However, the limited number of non-white participants, resulting from the absence of ethnic minority oversampling, prevented us from investigating ethnicity in our analysis.

## 5 Conclusions

Our findings demonstrate that receiving care indicates increased susceptibility to frailty and identifies individuals who are less likely to experience a reduction in their level of frailty. Household wealth emerges as an equally influential factor in predicting these transitions, highlighting that the risk of frailty for low-wealth individuals who *do not* receive any care is the same as the risk for high-wealth individuals who *do* receive care. As individuals receiving care are likely to be in poorer health than those who do not receive care, this result emphasises the stark inequalities in frailty outcomes between socio-economic groups. This care encompasses both unpaid and paid care. Interventions aimed at preventing frailty may be of greatest benefit to individuals who start to receive care and to those with lower levels of wealth. Unmet need for care does not appear to be strongly associated with changes in frailty, although this may be due to the small number of people reporting unmet needs.

## Funding

This research is funded through the National Institute for Health and Care Research (NIHR) Policy Research Unit in Older People and Frailty (funding reference PR-PRU-1217–2150). As of 01.01.24, the unit has been renamed to the NIHR Policy Research Unit in Healthy Ageing (funding reference NIHR206119). The views expressed are those of the author(s) and not necessarily those of the NIHR or the Department of Health and Social Care.

The sponsor had no role in the design and conduct of the study; in the collection, analysis, and interpretation of data; in the preparation of the manuscript; or in the review or approval of the manuscript.

## Declaration of competing interest

On behalf of all authors, the corresponding author states that there is no conflict of interest.

## Appendix Supplementary materials

Image, application 1
