# Supplementary material for: Differences in the risk of frailty based on care receipt, unmet care needs and socio-economic inequalities: A longitudinal analysis of the English Longitudinal Study of Ageing
Source: J Frailty Aging. 2025 Mar 7;14(2):100012. doi: 10.1016/j.tjfa.2025.100012 (PMC11959111; doi:10.1016/j.tjfa.2025.100012)
Supplement: Supplementary file 1 [file mmc1.docx]

**Supplementary material: *Differences in the risk of frailty based on care receipt, unmet care needs and socio-economic inequalities: a longitudinal analysis of the English Longitudinal Study of Ageing***

**Contents**

A1: Mortality data

A2: Frailty index deficits

A3: Variables used in the definition of Received Care

A4: Overview of sensitivity analyses

A5: Results of care receipt models with each set of covariates

A6: Results of unmet need for care models with each set covariates

A7: Comparison of care receipt and wealth

A8: Sensitivity analysis for receipt of care definition

A9: Sensitivity analysis for unmet need for care definition

A10: Sensitivity analysis for including year of birth as a covariate

A11 Sensitivity analysis: Subject characteristics

Appendix References

**A1: Mortality data**

Participants’ date of death data was obtained from three sources. A subset (21%) of ELSA participants known to have died before wave 6 is linked to end-of-life interviews which include their year of death [1]. The year of death is not provided for the remaining participants known to have died before wave 6, however, the wave of death is provided, allowing the year of death range for each participant to be narrowed down to 2-3 years. We used a uniform random distribution to assign a date of death (day and month) to each of these participants, bounded by the beginning and end of their year of death range. Year of death for participants known to have died in waves 6-9 was obtained by linking Office for National Statistics mortality data to ELSA participants. Again, the exact date of death (within the known year) was drawn from a uniform random distribution. We also used a uniform random distribution to generate dates of birth, as only the year of birth of ELSA participants was provided.

**A2: Frailty index deficits**

Deficits correspond to those in Supplementary Table 1 of Maharani et al [2].

| **Description** |
| --- |
| 1. Difficulty with walking 100 yards |
| 1. Difficulty sitting for about two hours |
| 1. Difficulty getting up from a chair after sitting for long periods |
| 1. Difficulty climbing several flights of stairs without resting |
| 1. Difficulty climbing one flight of stairs without resting |
| 1. Difficulty stooping, kneeling, or crouching |
| 1. Difficulty reaching or extending arms above shoulder level |
| 1. Difficulty pulling or pushing large objects like a living room chair |
| 1. Difficulty lifting or carrying weights over 10 pounds, like a heavy bag |
| 1. Difficulty picking up a 5p coin from a table |
| 1. Difficulty dressing, including putting on shoes and socks |
| 1. Difficulty walking across a room |
| 1. Difficulty bathing or showering |
| 1. Difficulty eating, such as cutting up your food |
| 1. Difficulty getting in or out of bed |
| 1. Difficulty using the toilet, including getting up or down |
| 1. Difficulty using a map to figure out how to get around in a strange place |
| 1. Difficulty preparing a hot meal |
| 1. Difficulty shopping for groceries |
| 1. Difficulty making telephone calls |
| 1. Difficulty taking medications |
| 1. Difficulty managing money, (e.g. paying bills and keeping track of expenses) |
| 1. Difficulty doing work around the house or garden |
| 1. Self-reported general health |
| 1. Whether respondent has felt depressed much of the time during the past week |
| 1. Whether respondent felt everything they did during the past week was an effort |
| 1. Whether respondent felt their sleep was restless much of the time during the past week |
| 1. Whether respondent was happy much of the time during the past week |
| 1. Whether respondent felt lonely much of the time during the past week |
| 1. Whether the respondent enjoyed life much of the time during the past week |
| 1. Whether respondent felt sad much of the time during the past week |
| 1. Whether respondent could not get going much of the time during the past week |
| 1. High blood pressure or hypertension (self-reported) |
| 1. Angina (self-reported) |
| 1. Heart attack (including MI or coronary thrombosis) (self-reported) |
| 1. Congestive heart failure (self-reported) 2. An abnormal heart rhythm (self-reported) |
| 1. Diabetes or high blood sugar (self-reported) |
| 1. A stroke (cerebral vascular disease) (self-reported) |
| 1. Chronic lung disease such as chronic bronchitis or emphysema (self-reported) |
| 1. Asthma (self-reported) |
| 1. Arthritis (including osteoarthritis, or rheumatism) (self-reported) |
| 1. Osteoporosis, sometimes called thin or brittle bones (self-reported) |
| 1. Cancer or a malignant tumour (excluding minor skin cancers) (self-reported) |
| 1. Parkinson's disease (self-reported) |
| 1. Any emotional, nervous or psychiatric problems (self-reported) |
| 1. Alzheimer's disease (self-reported) |
| 1. Dementia, organic brain syndrome, senility or any other serious memory impairment (self-reported) |
| 1. Self-reported eyesight function (while using lenses, if appropriate) |
| 1. Self-reported hearing function (while using hearing aid if appropriate) |
| 1. Whether respondent has fallen down at all /last year /last 2 years |
| 1. Whether respondent has fractured hip ever /in last 2 years |
| 1. Whether respondent has had joint replacement ever |
| 1. Whether respondent has had any pain whilst walking |
| 1. Identify today's date: day of month |
| 1. Identify today's date: month |
| 1. Identify today's date: year |
| 1. Identify the day of the week |
| 1. Immediate word recall (sample organized into quartiles) |
| 1. Delayed word recall (sample organized into quintiles) |

**A3: Variables used in the definition of Received Care**

Questions on receiving care associated with these activities have been included in each ELSA wave, although the questions were modified slightly after wave 6. Since wave 6, participants have been asked to only consider help received in the past month. No time period was specified in earlier waves, instead, participants were asked not to include difficulties they expected to last less than three months.

| Variables used in the definition of received care for the main analysis and sensitivity analysis. The main analysis treats received care as a binary variable, while the sensitivity analysis models it as a continuous variable with values 0-2. The value of the sensitivity analysis column relates to the level of care (no care = 0; low-level = 1; high-level = 2). ADL: Activity of Daily Living, IADL: Instrumental Activity of Daily Living. Meals on wheels and the use of a day centre are not considered ADLs or IADLs. | | | |
| --- | --- | --- | --- |
| **Care type** | **ADL/IADL** | **Main analysis** | **Sensitivity analysis** |
| Using the toilet, including getting up or down | ADL | Yes | 2 |
| Getting in and out of bed | ADL | Yes | 2 |
| Eating, such as cutting up food | ADL | Yes | 2 |
| Bathing or shower | ADL | Yes | 2 |
| Walking across a room | ADL | Yes | 2 |
| Dressing, including putting on shoes and socks | ADL | Yes | 2 |
| Shopping for groceries | IADL | Yes | 1 |
| Doing work around the house or garden | IADL | Yes | 1 |
| Managing money, such as paying bills and keeping track of expenses | IADL | Yes | 1 |
| Climbing several flights of stairs without resting | Mobility | Yes | 1 |
| Climbing one flight of stairs without resting | Mobility | Yes | 1 |
| Taking medication | IADL | Yes | 1 |
| Walking 100 yards | Mobility | Yes | 1 |
| Meals on wheels | - | No | 2 |
| Day centre | - | No | 1 |
| Did not receive care | - | No | 0 |

**A4: Overview of sensitivity analyses**

*Sensitivity analyses methods:*

Alternative, broader definitions of receipt of care and unmet need for care were included as a sensitivity analysis. The broader definition of received care added the use of meals on wheels and the use of a day centre in the past month. Care activities were also categorised as high or low-level care. Care for activities of daily living and meals on wheels were categorised as high-level care and help with instrumental activities of daily living, help climbing stairs without resting and use of a day centre were recorded as low-level care (Appendix A3). This was treated as an ordinal variable, with high-level care assigned a value of 2, low-level care 1, and no care 0. Questions on the use of meals on wheels and day centres were introduced to ELSA in wave 6, restricting this sensitivity analysis to waves 6-9 (2012-2019).

The broader definition of unmet need for care included participants who reported that their care ‘usually’, ‘sometimes’ or ‘hardly ever’ meets their needs (versus ‘always’ meets their needs). In the primary definition of unmet care, ‘usually’ was considered to mean the participant does not have an unmet need for care.

*Sensitivity analyses results:*

Results of multistate models with the broader definition of care receipt and unmet need for care are presented in Appendix A8 and A9. The broader definitions of care receipt (ELSA wave 6) and unmet need for care (ELSA wave 1) are shown in Appendix A11. Low-level care is required by 10.8% of ELSA wave 6 participants, and high-level care by 7.8% (Appendix A9) (these sum to a lower value than 21.1% which receive care reported in Table 1, as Table 1 reports data from ELSA wave 1). Using the broader definition of unmet need for care, 8.2% of ELSA wave 1 have an unmet need for care, compared to 2.6% in the primary definition (Table 1 and Appendix A11).

The hazard ratios follow similar patterns with both definitions of received care (Appendix A6). Increased care is associated with a greater risk of an increase in a person’s level of frailty and a decreased risk of reducing frailty.

Comparable hazard ratios are also present with the broader definition of unmet need for care (Appendix A9). The only significant changes between the broader definition and the narrower, main definition of unmet need for care are a greater hazard ratio for frail-to-prefrail for wealthier males and a greater hazard ratio for prefrail-to-frail for females with unmet need for care.

ELSA limits the precision of participants’ dates of birth and death. Precise dates of birth were randomly assigned, within the boundaries of the data provided (e.g., randomly selecting one day during a given year of birth). We confirmed that our results were not sensitive to the generated dates by regenerating the dates for five additional model fits of the best-fitting care receipt model (covariates: age, wealth, care receipt).

Year of birth was included as an additional covariate in the best-fitting care receipt model (covariates: age, wealth, care receipt, Table 2) to see whether including demographic change in populations improved model fit (Appendix A10). These models did not improve the fit (as measured by BIC), hence simpler models without birth year are favoured.

An interaction between wealth and care receipt was tested and found to be not significant.

Multistate models typically apply the Markov assumption to simplify model fitting. The Markov assumption asserts that the probability of a transition does not depend on the previous states occupied by a person, only the current state. Further models that relaxed the Markov assumption were investigated. In these models, the probability of transitioning from the current state additionally depended on whether the person had ever previously been frail or prefrail. However, the model fit did not improve.

*Sensitivity analyses discussion:*

Using a broader definition of unmet need for care produces greater hazard ratios for the prefrail-to-frail transition for females with unmet need for care. With the broader definition, care must ‘always’ meet a person’s need for them to have no unmet need for care. This threshold may disproportionately be met for those with few needs, a set of people who may be able to remain prefrail for a longer period than those with many care needs. The absence of this distinction among males may imply a variation in how 'usually' and 'always' are interpreted by males and females.

**A5: Results of care receipt models with each set of covariates**

| Table A5a: Transition hazard ratios for the receipt of care and wealth model. Wealth is categorised into quintiles, with quintile 1 being the least wealthy. It was not possible to accurately constrain the 95% confidence interval of robust-death for males due to a low number of transitions. Bayesian Information Criterion (BIC): 50 164 (Male) 61 117 (Female). CI: 95% Confidence interval. | | | | | | |
| --- | --- | --- | --- | --- | --- | --- |
| **Male** | **Age [CI]** | | **Receive care [CI]** | | **Wealth [CI]** | |
| Robust–Prefrail | 1.004 | [1.004-1.005] | 2.09 | [1.70-2.58] | 0.87 | [0.85-0.90] |
| Robust–Death | 1.007 | [1.005-1.009] | 0.17 | [-] | 0.81 | [0.70-0.94] |
| Prefrail–Robust | 0.997 | [0.996-0.997] | 0.47 | [0.39-0.55] | 1.16 | [1.13-1.20] |
| Prefrail–Frail | 1.003 | [1.003-1.004] | 2.56 | [2.27-2.90] | 0.82 | [0.78-0.85] |
| Prefrail–Death | 1.007 | [1.006-1.008] | 1.10 | [0.84-1.45] | 0.95 | [0.88-1.04] |
| Frail–Prefrail | 0.999 | [0.999-1.000] | 0.65 | [0.55-0.77] | 1.08 | [1.01-1.16] |
| Frail–Death | 1.005 | [1.005-1.006] | 1.04 | [0.86-1.26] | 1.02 | [0.96-1.09] |
| **Female** | | | | | | |
| Robust–Prefrail | 1.003 | [1.003-1.004] | 1.75 | [1.50-2.04] | 0.88 | [0.86-0.90] |
| Robust–Death | 1.008 | [1.006-1.010] | 0.88 | [0.14-5.59] | 0.97 | [0.81-1.16] |
| Prefrail–Robust | 0.996 | [0.996-0.996] | 0.48 | [0.42-0.54] | 1.12 | [1.09-1.15] |
| Prefrail–Frail | 1.003 | [1.003-1.004] | 2.26 | [2.05-2.48] | 0.83 | [0.80-0.86] |
| Prefrail–Death | 1.009 | [1.008-1.010] | 1.17 | [0.87-1.57] | 0.84 | [0.76-0.94] |
| Frail–Prefrail | 0.999 | [0.998-0.999] | 0.64 | [0.56-0.74] | 1.18 | [1.12-1.24] |
| Frail–Death | 1.006 | [1.005-1.006] | 1.13 | [0.92-1.40] | 1.01 | [0.95-1.07] |

| Table A5b: Transition hazard ratios for the receipt of care and education model. Education is split into three ordered categories: lower than secondary school (0), secondary school (1), and college or higher (2). BIC: 51 493 (Male), 63 151 (Female). CI: 95% Confidence interval. It was not possible to accurately constrain the robust-death transition for males in receipt of care due to the small number of recorded transitions. | | | | | | |
| --- | --- | --- | --- | --- | --- | --- |
| **Male** | **Age [CI]** | | **Receive care [CI]** | | **Education [CI]** | |
| Robust–Prefrail | 1.004 | [1.004-1.005] | 2.08 | [1.69-2.56] | 0.89 | [0.86-0.93] |
| Robust–Death | 1.007 | [1.005-1.009] | 0.26 | [-] | 1.07 | [0.82-1.38] |
| Prefrail–Robust | 0.997 | [0.997-0.998] | 0.46 | [0.38-0.54] | 1.18 | [1.12-1.24] |
| Prefrail–Frail | 1.003 | [1.003-1.004] | 2.62 | [2.32-2.95] | 0.86 | [0.81-0.92] |
| Prefrail–Death | 1.007 | [1.006-1.008] | 1.14 | [0.86-1.51] | 0.89 | [0.79-1.01] |
| Frail–Prefrail | 0.999 | [0.999-1.000] | 0.67 | [0.57-0.79] | 1.00 | [0.92-1.09] |
| Frail–Death | 1.005 | [1.005-1.006] | 0.91 | [0.77-1.09] | 0.99 | [0.91-1.07] |
| **Female** | | | | | | |
| Robust–Prefrail | 1.003 | [1.003-1.003] | 1.77 | [1.52-2.06] | 0.89 | [0.86-0.93] |
| Robust–Death | 1.008 | [1.005-1.010] | 0.72 | [0.08-6.11] | 0.76 | [0.58-1.00] |
| Prefrail–Robust | 0.996 | [0.996-0.997] | 0.48 | [0.43-0.55] | 1.09 | [1.04-1.14] |
| Prefrail–Frail | 1.003 | [1.003-1.004] | 2.23 | [2.03-2.44] | 0.85 | [0.81-0.90] |
| Prefrail–Death | 1.009 | [1.007-1.010] | 1.40 | [1.04-1.90] | 0.89 | [0.76-1.05] |
| Frail–Prefrail | 0.999 | [0.998-0.999] | 0.70 | [0.61-0.80] | 1.09 | [1.02-1.17] |
| Frail–Death | 1.006 | [1.005-1.006] | 0.85 | [0.73-1.01] | 0.95 | [0.88-1.03] |

| Table A5c: Transition hazard ratios of the receipt of care and area deprivation model. Area deprivation is categorised as quintiles, with quintile 1 being the most deprived. BIC: 51 237 (Male), 62 926 (Female). CI (95% Confidence interval). | | | | | | |
| --- | --- | --- | --- | --- | --- | --- |
| **Male** | **Age [CI]** | | **Receive care [CI]** | | **Deprivation [CI]** | |
| Robust–Prefrail | 1.004 | [1.004-1.005] | 2.10 | [1.70-2.58] | 0.91 | [0.89-0.94] |
| Robust–Death | 1.007 | [1.005-1.009] | 0.59 | [0.03-12.03] | 0.74 | [0.63-0.87] |
| Prefrail–Robust | 0.997 | [0.997-0.997] | 0.45 | 0.38-0.54] | 1.15 | [1.11-1.19] |
| Prefrail–Frail | 1.003 | [1.003-1.004] | 2.61 | [2.31-2.94] | 0.87 | [0.83-0.90] |
| Prefrail–Death | 1.007 | [1.006-1.008] | 1.13 | [0.85-1.50] | 0.97 | [0.90-1.06] |
| Frail–Prefrail | 0.999 | [0.999-1.000] | 0.67 | [0.56-0.79] | 1.05 | [0.99-1.11] |
| Frail–Death | 1.005 | [1.005-1.006] | 0.91 | [0.77-1.09] | 1.00 | [0.95-1.06] |
| **Female** | | | | | | |
| Robust–Prefrail | 1.003 | [1.003-1.004] | 1.76 | [1.51-2.05] | 0.92 | [0.89-0.94] |
| Robust–Death | 1.008 | [1.006-1.010 | 0.73 | [0.10-5.28] | 0.84 | [0.71-1.01] |
| Prefrail–Robust | 0.996 | [0.996-0.996] | 0.48 | [0.43-0.55] | 1.10 | [1.07-1.14] |
| Prefrail–Frail | 1.003 | [1.003-1.004 | 2.21 | [2.02-2.43] | 0.89 | [0.86-0.92] |
| Prefrail–Death | 1.009 | [1.008-1.010] | 1.35 | [1.01-1.81] | 0.97 | [0.88-1.08] |
| Frail–Prefrail | 0.998 | [0.998-0.999] | 0.69 | [0.60-0.79] | 1.11 | [1.06-1.16] |
| Frail–Death | 1.006 | [1.005-1.006] | 0.88 | [0.75-1.03] | 0.96 | [0.91-1.01] |

| Table A5d: Transition hazard ratios for the receipt of care and marital status model. Marital status is categorised as married (=1) or not married (=0). BIC: 51 501 (Male), 63 188 (Female). CI (95% Confidence interval). It was not possible to accurately constrain the robust-death transition for males in receipt of care due to the small number of recorded transitions. | | | | | | |
| --- | --- | --- | --- | --- | --- | --- |
| **Male** | **Age [CI]** | | **Receive care [CI]** | | **Marital status [CI]** | |
| Robust–Prefrail | 1.004 | [1.004-1.005] | 2.13 | [1.73-2.62] | 0.84 | [0.77-0.91] |
| Robust–Death | 1.007 | [1.005-1.009] | 0.41 | [-] | 0.57 | [0.37-0.88] |
| Prefrail–Robust | 0.997 | [0.997-0.998] | 0.44 | [0.37-0.52] | 1.29 | [1.16-1.44] |
| Prefrail–Frail | 1.003 | [1.003-1.004] | 2.69 | [2.39-3.04] | 0.75 | [0.66-0.84] |
| Prefrail–Death | 1.007 | [1.006-1.008] | 1.14 | [0.85-1.52] | 0.74 | [0.58-0.94] |
| Frail–Prefrail | 0.999 | [0.999-1.000] | 0.67 | [0.56-0.79] | 1.03 | [0.87-1.23] |
| Frail–Death | 1.005 | [1.005-1.006] | 0.91 | [0.77-1.09] | 0.93 | [0.80-1.08] |
| Female | | | | | | |
| Robust–Prefrail | 1.003 | [1.003-1.003] | 1.81 | [1.56-2.11] | 0.85 | 0.79-0.91 |
| Robust–Death | 1.008 | [1.005-1.010] | 0.71 | [0.08-6.58] | 0.68 | 0.42-1.09 |
| Prefrail–Robust | 0.996 | [0.996-0.997] | 0.48 | [0.42-0.54] | 1.13 | 1.03-1.23 |
| Prefrail–Frail | 1.003 | [1.003-1.003] | 2.26 | [2.06-2.48] | 0.77 | 0.70-0.84 |
| Prefrail–Death | 1.008 | [1.007-1.010] | 1.40 | [1.03-1.90] | 0.76 | 0.55-1.05 |
| Frail–Prefrail | 0.999 | [0.998-0.999] | 0.68 | [0.59-0.77] | 1.15 | 1.01-1.31 |
| Frail–Death | 1.006 | [1.005-1.007] | 0.84 | [0.72-1.00] | 1.13 | 0.97-1.33 |

**A6: Results of unmet need for care models with each set covariates**

| Table A6a: Transition hazard ratios of the unmet need for care model, covariates: age, unmet need for care, wealth. Wealth is split into five quintiles, with quintile 1 being the least wealthy. BIC: 43 818 (Male), 55 392 (Female). CI (95% Confidence interval). | | | | | | |
| --- | --- | --- | --- | --- | --- | --- |
| **Male** | **Age [CI]** | | **Unmet care [CI]** | | **Wealth [CI]** | |
| Robust–Prefrail | 1.005 | [1.004-1.005] | 0.76 | [0.45-1.29] | 0.87 | [0.85-0.90] |
| Robust–Death | 1.005 | [0.998-1.012] | 1.01 | [0.12-8.74] | 0.43 | [0.14-1.35] |
| Prefrail–Robust | 0.997 | [0.996-0.997] | 0.78 | [0.46-1.32] | 1.18 | [1.14-1.22] |
| Prefrail–Frail | 1.004 | [1.004-1.005] | 1.71 | [1.21-2.42] | 0.83 | [0.80-0.87] |
| Prefrail–Death | 1.002 | [0.999-1.004] | 1.03 | [0.85-1.24] | 1.01 | [0.80-1.27] |
| Frail–Prefrail | 0.998 | [0.998-0.999] | 1.02 | [0.94-1.10] | 1.05 | [0.98-1.12] |
| Frail–Death | 1.004 | [1.003-1.004] | 1.00 | [0.96-1.03] | 1.10 | [1.04-1.17] |
| **Female** | | | | | | |
| Robust–Prefrail | 1.003 | [1.003-1.004] | 0.78 | [0.58-1.06] | 0.88 | [0.86-0.90] |
| Robust–Death | 1.004 | [1.001-1.007] | 1.03 | [0.05-20.4] | 0.76 | [0.62-0.93] |
| Prefrail–Robust | 0.996 | [0.995-0.996] | 0.78 | [0.58-1.06] | 1.13 | [1.10-1.16] |
| Prefrail–Frail | 1.004 | [1.004-1.004] | 1.00 | [0.97-1.03] | 0.84 | [0.82-0.87] |
| Prefrail–Death | 1.007 | [1.003-1.011] | 1.05 | [0.06-17.0] | 0.37 | [0.16-0.88] |
| Frail–Prefrail | 0.998 | [0.998-0.999] | 1.01 | [0.97-1.05] | 1.15 | [1.10-1.21] |
| Frail–Death | 1.004 | [1.004-1.005] | 0.99 | [0.98-1.01] | 1.13 | [1.08-1.18] |

| Table A6b: Transition hazard ratios of the unmet need for care model, other covariates: age, education. Education is split into lower than secondary school, secondary school and college or higher. BIC: 44 917 (Male), 56 980 (Female). CI (95% Confidence interval). It was not possible to accurately constrain the robust-death transition for females with an unmet need for care due to the small number of recorded transitions. | | | | | | |
| --- | --- | --- | --- | --- | --- | --- |
| **Male** | **Age [CI]** | | **Unmet care [CI]** | | **Education [CI]** | |
| Robust–Prefrail | 1.004 | [1.004-1.005] | 0.75 | [0.46-1.24] | 0.90 | [0.86-0.94] |
| Robust–Death | 1.005 | [1.001-1.009] | 1.02 | [0.25-4.21] | 0.54 | [0.09-3.10] |
| Prefrail–Robust | 0.997 | [0.997-0.998] | 0.77 | [0.47-1.26] | 1.21 | [1.15-1.28] |
| Prefrail–Frail | 1.004 | [1.003-1.004] | 1.80 | [1.28-2.52] | 0.84 | [0.78-0.91] |
| Prefrail–Death | 1.001 | [0.998-1.004] | 1.03 | [0.82-1.28] | 0.96 | [0.38-2.41] |
| Frail–Prefrail | 0.999 | [0.998-0.999] | 1.02 | [0.94-1.10] | 1.00 | [0.91-1.10] |
| Frail–Death | 1.004 | [1.003-1.004] | 1.01 | [0.95-1.08] | 1.02 | [0.92-1.13] |
| **Female** | | | | | | |
| Robust–Prefrail | 1.003 | [1.003-1.004] | 0.76 | [0.57-1.02] | 0.89 | [0.86-0.92] |
| Robust–Death | 1.004 | [1.001-1.007] | 1.04 | [-] | 0.61 | [0.43-0.88] |
| Prefrail–Robust | 0.996 | [0.996-0.996] | 0.77 | [0.58-1.02] | 1.10 | [1.05-1.15] |
| Prefrail–Frail | 1.004 | [1.004-1.004] | 1.00 | [0.98-1.02] | 0.86 | [0.82-0.90] |
| Prefrail–Death | 1.003 | [1.001-1.006] | 1.34 | [0.27-6.59] | 0.51 | [0.32-0.80] |
| Frail–Prefrail | 0.998 | [0.998-0.999] | 1.01 | [0.97-1.04] | 1.11 | [1.04-1.19] |
| Frail–Death | 1.005 | [1.004-1.005] | 1.00 | [0.98-1.01] | 1.01 | [0.95-1.08] |

| Table A6c: Transition hazard ratios of the unmet need for care model, other covariates: age, area deprivation. Area deprivation is split into five quintiles, with quintile 1 being the most deprived. BIC: 44 686 (Male), 56 744 (Female). CI (95% Confidence interval). | | | | | | |
| --- | --- | --- | --- | --- | --- | --- |
| **Male** | **Age [CI]** | | **Unmet care [CI]** | | **Deprivation [CI]** | |
| Robust–Prefrail | 1.004 | [1.004-1.005] | 0.75 | [0.45-1.28] | 0.91 | [0.89-0.94] |
| Robust–Death | 1.002 | [0.996-1.008] | 1.01 | [0.06-17.16] | 0.45 | [0.15-1.40] |
| Prefrail–Robust | 0.997 | [0.996-0.997] | 0.78 | [0.46-1.31] | 1.16 | [1.12-1.21] |
| Prefrail–Frail | 1.004 | [1.004-1.005] | 1.77 | [1.27-2.48] | 0.88 | [0.84-0.92] |
| Prefrail–Death | 1.001 | [0.999-1.004] | 1.02 | [0.86-1.23] | 0.95 | [0.69-1.31] |
| Frail–Prefrail | 0.998 | [0.998-0.999] | 1.02 | [0.94-1.10] | 1.03 | [0.97-1.09] |
| Frail–Death | 1.004 | [1.003-1.004] | 1.00 | [0.97-1.03] | 1.07 | [1.01-1.13] |
| **Female** | | | | | | |
| Robust–Prefrail | 1.003 | [1.003-1.004] | 0.74 | [0.55-0.98] | 0.92 | [0.90-0.94] |
| Robust–Death | 1.002 | [0.999-1.006] | 1.03 | [0.12-8.78] | 0.68 | [0.54-0.86] |
| Prefrail–Robust | 0.996 | [0.995-0.996] | 0.74 | [0.55-0.98] | 1.11 | [1.08-1.14] |
| Prefrail–Frail | 1.004 | [1.004-1.005] | 1.02 | [0.98-1.05] | 0.89 | [0.87-0.92] |
| Prefrail–Death | 1.003 | [0.996-1.010] | 1.05 | [0.01-141] | 0.27 | [0.04-1.98] |
| Frail–Prefrail | 0.998 | [0.998-0.998] | 1.01 | [0.97-1.05] | 1.11 | [1.06-1.15] |
| Frail–Death | 1.004 | [1.004-1.005] | 0.99 | [0.98-1.01] | 1.05 | [1.01-1.09] |

| Table A6d: Transition hazard ratios of the unmet need for care model, other covariates: age, marital status. Marital status is categorised as married (=1) or not married (=0). BIC: 44 990 (Male), 57 062 (Female). CI (95% Confidence interval). | | | | | | |
| --- | --- | --- | --- | --- | --- | --- |
| **Male** | **Age [CI]** | | **Unmet care [CI]** | | **Marital status [CI]** | |
| Robust–Prefrail | 1.004 | [1.004-1.005] | 0.76 | [0.46-1.26] | 0.84 | [0.77-0.92] |
| Robust–Death | 1.004 | [0.998-1.011] | 1.02 | [0.39-2.66] | 0.46 | [0.08-2.71] |
| Prefrail–Robust | 0.997 | [0.997-0.997] | 0.78 | [0.48-1.28] | 1.26 | [1.13-1.41] |
| Prefrail–Frail | 1.004 | [1.003-1.004] | 1.76 | [1.26-2.45] | 0.80 | [0.70-0.93] |
| Prefrail–Death | 1.001 | [0.996-1.006] | 1.03 | [0.82-1.29] | 0.69 | [0.10-4.73] |
| Frail–Prefrail | 0.999 | [0.998-0.999] | 1.00 | [0.96-1.04] | 0.92 | [0.77-1.10] |
| Frail–Death | 1.004 | [1.003-1.004] | 1.01 | [0.96-1.07] | 1.01 | [0.84-1.21] |
| **Female** | | | | | | |
| Robust–Prefrail | 1.003 | [1.003-1.003] | 0.75 | [0.56-1.01] | 0.85 | [0.80-0.91] |
| Robust–Death | 1.005 | [1.002-1.008] | 1.03 | [0.02-43.8] | 0.62 | [0.32-1.20] |
| Prefrail–Robust | 0.996 | [0.996-0.996] | 0.76 | [0.56-1.01] | 1.07 | [0.98-1.16] |
| Prefrail–Frail | 1.004 | [1.004-1.004] | 1.01 | [0.98-1.03] | 0.81 | [0.75-0.88] |
| Prefrail–Death | 1.003 | [1.000-1.005] | 1.04 | [0.17-6.44] | 0.49 | [0.24-0.98] |
| Frail–Prefrail | 0.998 | [0.998-0.999] | 1.01 | [0.97-1.06] | 1.04 | [0.92-1.18] |
| Frail–Death | 1.005 | [1.004-1.005] | 1.00 | [0.98-1.01] | 1.17 | [1.03-1.33] |

**A7: Comparison of care receipt and wealth**

| Table A7: (a) Comparison of hazard ratios between (i) care receipt versus no care receipt and (ii) the wealthiest versus the least wealthy quintile. Note that the hazard ratio for the wealth quintiles is inverted compared to other hazard ratios in this manuscript (which show wealthier quintiles relative to less wealthy quintiles) to facilitate comparison with the care receipt hazard ratios. It was not possible to accurately constrain the 95% confidence interval of robust-death for males due to a low number of transitions. CI: 95% Confidence Interval. | | | | |
| --- | --- | --- | --- | --- |
| **Male** | **Receive care relative to**  **No care [CI]** | | **Least wealth relative to**  **Most wealth [CI]** | |
| Robust–Prefrail | 2.09 | [1.70-2.58] | 1.73 | [1.56-1.93] |
| Robust–Death | 0.17 | [-] | 2.32 | [1.40-5.35] |
| Prefrail–Robust | 0.47 | [0.39-0.55] | 0.54 | [0.48-0.63] |
| Prefrail–Frail | 2.56 | [2.27-2.90] | 2.25 | [1.90-2.74] |
| Prefrail–Death | 1.10 | [0.84-1.45] | 1.20 | [0.89-1.76] |
| Frail–Prefrail | 0.65 | [0.55-0.77] | 0.73 | [0.57-0.98] |
| Frail–Death | 1.04 | [0.86-1.26] | 0.94 | [0.76-1.16] |
| **Female** | | | | |
| Robust–Prefrail | 1.75 | [1.50-2.04] | 1.65 | [1.50-1.83] |
| Robust–Death | 0.88 | [0.14-5.59] | 1.13 | [0.64-3.22] |
| Prefrail–Robust | 0.48 | [0.42-0.54] | 0.63 | [0.56-0.71] |
| Prefrail–Frail | 2.26 | [2.05-2.48] | 2.15 | [1.87-2.52] |
| Prefrail–Death | 1.17 | [0.87-1.57] | 1.98 | [1.37-3.33] |
| Frail–Prefrail | 0.64 | [0.56-0.74] | 0.52 | [0.43-0.65] |
| Frail–Death | 1.13 | [0.92-1.40] | 0.98 | [0.78-1.29] |

**Appendix A8: Sensitivity analysis for receipt of care definition**

| Table A8: Sensitivity analysis for receipt of care definition. Transition hazard ratios provided. The broader definition additionally includes the use of meals on wheels and the use of a day centre in the past month (a). Receipt of care is split into three ordinal categories indicating level of care: none, low-level and high-level (c.f. No or Yes used in the main definition). Results are split by gender. This analysis is modelled on ELSA waves 6-9 only, as this data is not available for earlier waves. To allow comparison with the main definition of care receipt (responses: no or yes), hazard ratios using the main definition with waves 6-9 only are also provided (b). It was not possible to accurately constrain the robust-death in receipt of care due to the small number of recorded transitions. CI: 95% Confidence Interval. | | | | | | | | |
| --- | --- | --- | --- | --- | --- | --- | --- | --- |
| a: Receipt of care: none, low-level, high-level. | | | | | | | | |
|  | **Male** | | | | **Female** | | | |
| **Transition** | **Level of care [CI]** | | **Wealth [CI]** | | **Level of care [CI]** | | **Wealth [CI]** | |
| Robust–Prefrail | 1.48 | [1.11-1.97] | 0.87 | [0.83-0.91] | 1.35 | [1.04-1.75] | 0.88 | [0.84-0.92] |
| Robust–Death | 1.60 | [0.24-10.7] | 0.97 | [0.70-1.35] | 0.32 | [0.00-23.5] | 0.94 | [0.70-1.28] |
| Prefrail–Robust | 0.55 | [0.43-0.70] | 1.20 | [1.13-1.27] | 0.45 | [0.37-0.55] | 1.15 | [1.10-1.21] |
| Prefrail–Frail | 1.72 | [1.51-1.96] | 0.83 | [0.76-0.89] | 2.00 | [1.79-2.23] | 0.83 | [0.78-0.88] |
| Prefrail–Death | 1.06 | [0.77-1.46] | 0.82 | [0.71-0.95] | 0.87 | [0.57-1.33] | 0.88 | [0.75-1.03] |
| Frail–Prefrail | 0.60 | [0.50-0.71] | 1.18 | [1.05-1.33] | 0.59 | [0.51-0.69] | 1.22 | [1.12-1.33] |
| Frail–Death | 1.31 | [1.07-1.59] | 1.06 | [0.96-1.18] | 1.79 | [1.45-2.21] | 0.99 | [0.89-1.10] |
| b: Receipt of care: No or Yes. | | | | | | | | |
|  | **Male** | | | | **Female** | | | |
| **Transition** | **Received care [CI]** | | **Wealth [CI]** | | **Received care [CI]** | | **Wealth [CI]** | |
| Robust–Prefrail | 1.93 | [1.11-3.34] | 0.87 | [0.83-0.91] | 1.68 | [1.13-2.51] | 0.88 | [0.84-0.92] |
| Robust–Death | 0.70 | [-] | 0.97 | [0.69-1.37] | 0.15 | [-] | 0.94 | [0.69-1.27] |
| Prefrail–Robust | 0.38 | [0.25-0.56] | 1.20 | [1.13-1.27] | 0.33 | [0.25-0.44] | 1.15 | [1.09-1.21] |
| Prefrail–Frail | 2.58 | [2.07-3.22] | 0.82 | [0.76-0.89] | 2.74 | [2.33-3.23] | 0.82 | [0.77-0.87] |
| Prefrail–Death | 1.02 | [0.56-1.85] | 0.83 | [0.72-0.96] | 0.84 | [0.43-1.65] | 0.88 | [0.74-1.05] |
| Frail–Prefrail | 0.45 | [0.33-0.60] | 1.17 | [1.04-1.32] | 0.51 | [0.41-0.64] | 1.21 | [1.11-1.31] |
| Frail–Death | 1.40 | [0.96-2.05] | 1.06 | [0.95-1.17] | 1.83 | [1.22-2.73] | 0.99 | [0.90-1.10] |

**Appendix A9: Sensitivity analysis for unmet need for care definition**

| Table A9: Sensitivity analysis for unmet need for care definition. Transition hazard ratios provided. In this alternative, broader definition (a), unmet care need is where older people who report that their care ‘hardly ever’, ‘sometimes’ or ‘usually’ meets their needs are considered to have an unmet need for care. In the main analysis, (b), a person who reported that their care ‘usually’ meets their needs was not considered to have an unmet need. Results are split by gender. CI: 95% Confidence Interval. | | | | | | | | |
| --- | --- | --- | --- | --- | --- | --- | --- | --- |
| **a: Unmet need for care when care ‘hardly ever’, ‘sometimes’ or ‘usually’ meets participants’ needs** | | | | | | | | |
|  | **Male** | | | | **Female** | | | |
| **Transition** | **Unmet care [CI]** | | **Wealth** | | **Unmet care [CI]** | | **Wealth** | |
| Robust–Prefrail | 0.73 | [0.55-0.98] | 0.87 | [0.85-0.89] | 0.84 | [0.71-0.99] | 0.89 | [0.87-0.91] |
| Robust–Death | 1.02 | [0.20-5.18] | 0.21 | [0.08-0.53] | 1.01 | [0.73-1.41] | 0.76 | [0.61-0.93] |
| Prefrail–Robust | 0.74 | [0.56-0.99] | 1.15 | [1.11-1.19] | 0.82 | [0.70-0.96] | 1.15 | [1.12-1.18] |
| Prefrail–Frail | 2.10 | [1.78-2.47] | 0.87 | [0.83-0.91] | 1.37 | [1.22-1.54] | 0.86 | [0.84-0.89] |
| Prefrail–Death | 1.02 | [0.90-1.16] | 1.04 | [0.86-1.25] | 1.00 | [0.72-1.38] | 0.50 | [0.34-0.74] |
| Frail–Prefrail | 1.15 | [0.95-1.40] | 1.27 | [1.19-1.36] | 1.02 | [0.97-1.06] | 1.18 | [1.13-1.24] |
| Frail–Death | 0.99 | [0.97-1.01] | 1.11 | [1.05-1.18] | 0.99 | [0.98-1.01] | 1.09 | [1.04-1.14] |
| **b: Unmet need for care when care ‘hardly ever’ or ‘sometimes’ meets participants’ needs** | | | | | | | | |
|  | **Male** | | | | **Female** | | | |
| **Transition** | **Unmet care [CI]** | | **Wealth** | | **Unmet care [CI]** | | **Wealth** | |
| Robust–Prefrail | 0.76 | [0.45-1.29] | 0.87 | [0.85-0.90] | 0.78 | [0.58-1.06] | 0.88 | [0.86-0.90] |
| Robust–Death | 1.01 | [0.12-8.74] | 0.43 | [0.14-1.35] | 1.03 | [0.05-20.4] | 0.76 | [0.62-0.93] |
| Prefrail–Robust | 0.78 | [0.46-1.32] | 1.18 | [1.14-1.22] | 0.78 | [0.58-1.06] | 1.13 | [1.10-1.16] |
| Prefrail–Frail | 1.71 | [1.21-2.42] | 0.83 | [0.80-0.87] | 1.00 | [0.97-1.03] | 0.84 | [0.82-0.87] |
| Prefrail–Death | 1.03 | [0.85-1.24] | 1.01 | [0.80-1.27] | 1.05 | [0.06-17.0] | 0.37 | [0.16-0.88] |
| Frail–Prefrail | 1.02 | [0.94-1.10] | 1.05 | [0.98-1.12] | 1.01 | [0.97-1.05] | 1.15 | [1.10-1.21] |
| Frail–Death | 1.00 | [0.96-1.03] | 1.10 | [1.04-1.17] | 0.99 | [0.98-1.01] | 1.13 | [1.08-1.18] |

**A10: Sensitivity analysis for including year of birth as a covariate**

| Table A10: Transition hazard ratios of the receipt of care model, other covariates: age, wealth and birth year. Birth year is relative to the mean year of birth. Bayesian Information Criterion (BIC): 50 215 (Male), 61 306 (Female). CI: 95% Confidence Interval. | | | | | | | | |
| --- | --- | --- | --- | --- | --- | --- | --- | --- |
| **Male** | **Age [CI]** | | **Received care [CI]** | | **Wealth [CI]** | | **Birth year [CI]** | |
| Robust–Prefrail | 1.003 | [1.002-1.004] | 1.97 | [1.59-2.43] | 0.87 | [0.85-0.90] | 0.98 | [0.97-0.99] |
| Robust–Death | 1.002 | [0.998-1.006] | 0.85 | [0.14-5.10] | 0.78 | [0.67-0.92] | 0.94 | [0.90-0.98] |
| Prefrail–Robust | 0.997 | [0.996-0.997] | 0.46 | [0.39-0.55] | 1.17 | [1.13-1.21] | 1.00 | [0.99-1.01] |
| Prefrail–Frail | 1.003 | [1.002-1.005] | 2.54 | [2.25-2.87] | 0.82 | [0.78-0.85] | 1.00 | [0.99-1.01] |
| Prefrail–Death | 1.003 | [1.001-1.005] | 1.04 | [0.79-1.37] | 0.96 | [0.89-1.05] | 0.95 | [0.93-0.97] |
| Frail–Prefrail | 0.999 | [0.998-1.001] | 0.65 | [0.55-0.77] | 1.08 | [1.01-1.16] | 1.00 | [0.98-1.02] |
| Frail–Death | 1.004 | [1.003-1.005] | 1.02 | [0.84-1.23] | 1.02 | [0.96-1.08] | 0.98 | [0.97-1.00] |
| **Female** | | | | | | | | |
| Robust–Prefrail | 1.002 | [1.001-1.002] | 1.66 | [1.42-1.94] | 0.88 | [0.86-0.90] | 0.98 | [0.97-0.99] |
| Robust–Death | 1.004 | [0.999-1.008] | 1.02 | [0.21-4.90] | 0.98 | [0.82-1.17] | 0.96 | [0.91-1.01] |
| Prefrail–Robust | 0.996 | [0.995-0.997] | 0.47 | [0.42-0.54] | 1.12 | [1.09-1.16] | 1.00 | [0.99-1.01] |
| Prefrail–Frail | 1.003 | [1.002-1.004] | 2.25 | [2.05-2.48] | 0.83 | [0.80-0.86] | 0.99 | [0.98-1.00] |
| Prefrail–Death | 1.005 | [1.002-1.007] | 1.02 | [0.75-1.37] | 0.84 | [0.76-0.94] | 0.95 | [0.92-0.97] |
| Frail–Prefrail | 0.997 | [0.996-0.998] | 0.64 | [0.56-0.73] | 1.17 | [1.12-1.23] | 0.98 | [0.97-1.00] |
| Frail–Death | 1.006 | [1.005-1.007] | 1.11 | [0.91-1.35] | 1.01 | [0.95-1.07] | 1.00 | [0.98-1.01] |

**A11 Sensitivity analysis: Subject characteristics**

| **Characteristic** | **n** | **(%)** |
| --- | --- | --- |
| **Receipt of care (wave 6)** | | |
| No | 9651 | 81.4 |
| Low-level | 1277 | 10.8 |
| High-level | 927 | 7.8 |
| **Unmet need for care (wave 1)** | | |
| No | 8707 | 91.8 |
| Yes | 779 | 8.2 |

In the sensitivity analysis, an alternative, broader definition of receipt of care is used. The broader definition of received care added the use of meals on wheels and the use of a day centre in the past month, to the activities of daily living and instrumental activities of daily living. Care activities were also categorised as high or low-level care in the sensitivity analysis (compared to a binary yes/no for receipt of care in the main analysis). All activities of daily living, along with meals on wheels were categorised as high-level care and all instrumental activities of daily living, help climbing stairs without resting and use of a day centre were recorded as low-level care. The activities of daily living and instrumental activities of daily living are listed In Appendix A3.

**Appendix References**

1. Crawford, R. and P. Mei, *An overview of the ELSA ‘End of Life’ data*. 2018, The Institute for Fiscal Studies: <https://ifs.org.uk/sites/default/files/output_url_files/R144.pdf>.

2. Maharani, A., et al., *Household wealth, neighbourhood deprivation and frailty amongst middle-aged and older adults in England: a longitudinal analysis over 15 years (2002-2017).* Age Ageing, 2023. **52**(3).
